# Static lung volumes and diffusion capacity in adults 30 years after being diagnosed with asthma

**DOI:** 10.1186/s40733-022-00086-4

**Published:** 2022-08-04

**Authors:** Conrad Uldall Becker Schultz, Oliver Djurhuus Tupper, Charlotte Suppli Ulrik

**Affiliations:** 1 Department of Respiratory Medicine, Copenhagen University Hospital-Hvidovre, Hvidovre, Denmark; 2 Institute of Clinical Medicine, University of Copenhagen, Copenhagen, Denmark

**Keywords:** Asthma, Hyperinflation, Gas transfer, Airway resistance, Body plethysmography

## Abstract

**Background:**

Long-term follow-up studies of adults with well-characterized asthma are sparse. We aimed to explore static lung volumes and diffusion capacity after 30 + years with asthma.

**Methods:**

A total of 125 adults with an objectively verified diagnosis of asthma between 1974–1990 at a Danish respiratory outpatient clinic completed a follow-up visit 2017–19. All participants (age range 44–88 years) completed a comprehensive workup and were, based on these assessments, classified as having either active asthma or being in complete remission. The examination program included measurements of static lung volumes and diffusion capacity.

**Results:**

Participants with active asthma were hyperinflated (residual volume/total lung capacity ratio 0.43, 95% CI 0.41—0.45) (RV/TLC ratio) compared with those in remission (RV/TLC ratio 0.38, 95% CI 0.36—0.41) (*p* < 0.03). A tendency towards higher diffusion capacity per liter lung volume was seen in participants with active asthma (KCO 100% predicted, 95% CI 97—104) compared with those in remission (KCO 94% pred., 95% CI 89—99) (*P* = 0.10). Longer asthma duration was associated with a higher KCO 0.47% pred./year (95% CI 0.14—0.80), adjusted for age and smoking. Patients on GINA step 4 and 5 treatment were more hyperinflated ($\Delta$ RV 14% pred., 95% CI 3—27) and had higher airway resistance (mean 53% pred., 95% CI 9—97) than participants on lower GINA steps. Patients with uncontrolled disease had substantially higher airway resistance (72% pred. 95% CI 20—124) than well-controlled patients.

**Conclusion:**

Thirty years after a confirmed diagnosis of asthma, those continuing to have active asthma and those having severe asthma, have higher diffusion capacity and more hyperinflation than patients in remission.

## Background

Asthma is a common inflammatory airway disease characterized by variable airflow limitation and airway hyperresponsiveness often regarded as an allergy-related disease with onset in childhood and associated with a good prognosis [1]. However, for some asthma persists into adulthood and some have disease onset in adulthood [2]. Asthma in adulthood rarely remits [3]. While dynamic spirometry, symptom questionnaires and exhaled fractional nitric oxide are an integrated aspect of asthma management, static lung volumes and diffusion capacity, likely due to availability and time constraints, does not hold the same place. Yet, recent studies suggest an important clinical impact of abnormal static volumes [4].

The diffusion capacity, also called the transfer factor, measures the capacity to transfer gas from alveolar spaces into the alveolar-capillary blood [5]. There is conflicting evidence in the literature about whether the diffusion capacity for carbon monoxide (DLCO) is elevated in asthma. Meisner and Hugh-Jones [6] found values for the diffusion constant (KCO) above predicted values in 16 of 23 measurements in nine patients. In addition, Ogilvie, C [7]. also observed diffusion capacities among asthma patients exceeding the predicted values. It was reported that 11 out of 56 patients had a DLCO exceeding 130 percent of predicted. On the other hand, Cotes et al. [8] published studies that did not show any differences between patients with asthma and controls regarding diffusion capacity. De Weger et al. [9] demonstrated that both residual volume/total lung capacity ratio (RV/TLC) and RV% pred. are relevant indicators of hyperinflation in relation to patient-related outcomes, additionally TLC is another indicator [10]. However, it is not precisely clarified which parameter that best reflects hyperinflation. Hyperinflation is important as it is negatively associated with health and activity [4]. A predominant factor in decreased airway caliber and premature airway closure appears to be asthmatic airway inflammation. The decreased airway caliber leads to increased airway resistance of the more proximal bronchioles and bronchi. Since this airway resistance cannot be overcome and airways close prematurely, the air gets trapped in the lung’s distal segments, this in turn leads to hyperinflation [11–15].

Progressive decline in lung function and the relationship between repeated exacerbations and accelerated loss of lung function have been well recognized in patients with asthma [16]. Nevertheless, many questions are to be answered regarding this progressive decline, not least regarding underlying pathophysiology [17]. We, therefore, aimed to examine possible hyperinflation and levels of diffusion capacity among patients with active asthma compared with individuals in remission 30 years after an objectively verified diagnosis of asthma.

## Methods

The present study is a secondary cross-sectional examination of a cohort of individuals with well-characterized asthma. The cohort will be referred to as the treatable traits in the asthma (TRAIL) cohort. All participants provided informed consent. The study was approved by the ethical committee for the Capital Region of Denmark (H-17025043), the regional data safety committee for the capital region of Denmark (P-2019–712), and The Danish Data Protection Agency (2013–41-2618).

Previously, the patient cohort and methods have been described by Ulrik et al. [18] Patients included in the cohort, were all > 13 years at inclusion. The entire cohort consists of all patients at Frederiksberg Respiratory and Allergy Clinic diagnosed with and followed for asthma between 1974–1990. Patients were diagnosed with asthma if they had a typical history and if one or more of the following tests were positive: Forced expiratory volume in the first second (FEV_1_) -reversibility, diurnal variability in peak expiratory flow (PEF) rate or positive histamine provocation test. The typical case history at baseline consisted of cough triggered by exercise, chest tightness, episodes of wheezing or attacks of breathlessness, triggered by exercise, exposure to allergens, irritants, and/or respiratory infections.

1. FEV_1_–reversibility > 15% (and an absolute increase of > 150 ml) after a standard dose of short-acting β_2_-agonist (SABA) or oral corticosteroid (30 mg/d) for 14 days [19].
2. Diurnal variability in peak flow expiratory flow (PEF) rate > 20% and absolute variation > 100 L/min [20, 21].
3. Positive histamine provocation test, with a concentration of histamine that results in a 20% drop in FEV_1_ (PC_20_) ≤ 8 mg/ml [22].

All still living persons from the TRAIL cohort were invited to an examination at Hvidovre Hospital by letter. If no response was received during the first month a reminder was sent out. The participants were all initially asked whether they had asthma, previously had asthma, or if they had never had asthma. The visit consisted of a comprehensive examination of their current asthma status [3]:

1. Questionnaires, GINA symptoms control.
2. Current inhaler medication (cross-referenced with currently prescribed medication in the Danish common medicine card).
3. Spirometry and β_2_-reversibility. Significant reversibility was defined as an absolute increase in FEV_1_ of ≥ 200 ml and a ≥ 12% increase in relative volume after 0,4 mg salbutamol [23, 24].
4. Fractional exhaled Nitric Oxide (FeNO) was measured with EcoMedics CLD 88sp analyzer and DENOX 88. The mean value of the two measurements (flowrate 50 ml/min) was recorded [25].
5. Bronchial challenge test. Participants inhaled stepwise accumulating doses of mannitol until either: 635 mg was inhaled with no significant changes in FEV_1_, a 15% drop in FEV_1_ from the baseline value or a 10% drop between two doses. A positive test (PD15 ≤ 635 mg) was defined as airway hyperresponsiveness (AHR) [26].
6. Body plethysmography and diffusion capacity. Body plethysmography was used for measuring both dynamic and static lung volumes. This included both functional residual capacity (FRC), total lung capacity (TLC), residual volume (RV), vital capacity (VC), total airway resistance (sR_aw_), intrathoracic gas volume (ITGV), inspiratory capacity (IC), and expiratory reserve volume (ERV). The transfer factor for carbon monoxide also referred to as the diffusion capacity (DLCO) was measured by the single breath method (in mmol x min^−1^ × kPa^−1^) together with the diffusion constant KCO (DLCO/alveolar volume). The single breath method involves inhalation of carbon monoxide and a breath-hold of 8–10 s during which time the CO continuously moves from the alveoli into the blood [27]. Body plethysmography uses Boyle’s law to determine lung volumes. After determining FRC the ERV, VC and IC are measured, which allows the calculation of RV and TLC [28]. Reference values according to ECSC2005 [29].

The diagnostic procedures and measurements were performed according to current guidelines.

### Remission of asthma

Complete asthma remission was defined as the following:

1. No asthma symptoms within the last year (wheeze, chest tightness, dyspnea, cough, or sputum).
2. No currently prescribed and self-reported use of asthma medication within the last year.
3. No bronchodilator reversibility.
4. FeNO < 50 ppb [30].
5. No AHR.
6. Spirometry with FEV_1_ ≥ 80% pred. and FEV1/FVC ratio ≥ 0.70.

### Asthma control and treatment intensity

We separately evaluated the severity based on two surrogate markers at baseline and follow-up [31].

- GINA 2017 symptom control level consists of four yes or no questions: (1) Daytime symptoms more than twice/week? (2) Any night waking due to asthma? (3) Reliever needed more than twice/week (4) Any activity limitation due to asthma? The level of asthma control is then based on the number of questions answered yes:
  1. Well-controlled: 0.
  2. Partly controlled: 1 – 2.
  3. Uncontrolled 3 – 4.

The patients with active asthma were separated into groups based on current inhaler prescription, with mild (GINA step 1–2), moderate (GINA step 3) and severe (GINA step 4–5) asthma.

### Statistics

Patient characteristics were compared for the groups with and without active asthma using student’s t-test, Mann–Whitney U-test, and Fisher’s exact test. Patient characteristics for GINA treatment step and symptom control were compared using one-way ANOVA, Kruskal–Wallis, and Fisher’s exact test. We examined differences in static lung volumes (TLC and RV), DLCO, KCO, and sR_aw_ by t-test for groups with and without active asthma, linear regression for asthma duration, and ANOVA for GINA treatment level and GINA symptom control. For the difference in DLCO based on asthma duration, we adjusted for age and tobacco exposure (packyears). Binomial logistic regression analyses were performed to examine factors associated with air trapping and hyperinflation, both are inconsistently defined in the literature, we defined it as RV/TLC > 0.4, RV > 120% or TLC > 120% predicted) [9, 10]. Statistical analyses were performed using SAS Enterprise Guide, version 7.15 (SAS Institute Inc. Cary, NC, USA). A two-tailed *p*-value < 0.05 was determined as significant.

## Results

A total of 125 patients (of 616 alive) with a previous objectively verified diagnosis of asthma inclusion participated in the cross-sectional examination and were therefore included in the present analyses.

Patients were aged 44—88 at time of the examination. Due to physical limitations, three (static volumes) and five (diffusion capacity) patients, respectively, did not complete all measurements of lung function parameters. A total of 103 out of the 122 were classified as having active asthma and 19 as having asthma in complete remission. No significant differences in sex (*p* = 0.21), age (*p* = 0.25), height (*p* = 0.74) or weight (*p* = 0.86) were seen between those having active asthma and those being in remission at the examinations. Patient characteristics are summarized in Table 1. Patients in remission had significantly higher FEV_1_/FVC ratio (0.73, 95% CI 0.69 – 0.75) compared to participants with active asthma (0.65, 95% CI 0.64 – 0.67) (*p* = 0.0002).

**Table 1:** Patient characteristics in the TRAIL cohort according to asthma status at the follow-up visit

Variables	*Active asthma (n* = *106)*	*Asthma in complete remission (n* = *19)*	*P*-value
Age, yr	62.1 $\pm$ 8.4	59.4 $\pm$ 9.5	0.2034
Female/Male sex, n	61/45	14/5	0.2136
Height, cm	172.0 $\pm$ 9.1	172.8 $\pm$ 7.5	0.7049
Weight, kg	79.5 $\pm$ 15.9	78.7 $\pm$ 17.1	0.8555
BMI, kg/m^2^	26.0 (23.7—28.6)	26.2 (22.9—29.8)	0.7258
Smokers, n (%)	77 (62%)	14 (74%)	1.0000
Smoking, pack yr	26.8 $\pm$ 12.2	14.1 $\pm$ 61	0.540
FVC % pred	100.2 $\pm$ 15.7	106.3 $\pm$ 15.0	0.1199
FEV_1_% pred	83.9 $\pm$ 19.17	98.5 $\pm$ 17.8	0.0025
FEV_1_/FVC ratio	0.65 $\pm$ 0.09	0.73 $\pm$ 0.06	0.0002
TLC% pred	105.4 $\pm$ 12.0	105.7 $\pm$ 11.5	0.9045
DLCO %pred	87.7 $\pm$ 17.3	84.7 $\pm$ 11.6	0.3574
DLCO/VA %pred	100.4 $\pm$ 16.1	93.9 $\pm$ 10.3	0.0324
RV % pred	123.7 $\pm$ 26.7	111.8 $\pm$ 21.6	0.0713
RV/TLC ratio	0.43 $\pm$ 0.09	0.38 $\pm$ 0.06	0.0045
sR_aw_ % pred	145.7 $\pm$ 96.5	117.0 $\pm$ 77.3	0.2228

Values are given as mean $\pm$ standard deviation, for BMI median (25^th^-75^th^ percentile)

*FVC* Forced vital capacity, *FEV*_*1*_ Forced expiratory volume after one second, *TLC* Total lung capacity, *DLCO SB* total diffusing capacity for carbon monoxide, *DLCO/VA* diffusing capacity when adjusted for volume, *RV* Residual volume, *sR*_*aw*_ Total airway resistance. N = numbers of patients with either active asthma or patients with asthma in remission. 3 (active asthma) persons could not complete static lung measurement and 5 persons (4 active asthma and 1 in remission) could not complete diffusion capacity

### Static lung volumes

No significant difference was observed in TLC, expressed as a percentage of predicted value (%pred.), between participants with active asthma compared with those in complete remission. However, we found that participants with active asthma (RV/TLC ratio 0.43, 95% CI 0.41 – 0.45) had more air trapping compared with participants with asthma in remission (RV/TLC ratio 0.38, 95% CI 0.36 – 0.41) (*p* = 0.026). Patients with active asthma also had higher residual volumes (RV% pred. 123.7, 95% CI 118.4 – 128.4) compared with participants in remission (RV% pred. 111.8, 95% CI 101.4 – 122.2) (*p* = 0.04). The comparison between the two groups is summarized in Table 1. A total of 25 (23.6%) persons with active asthma were hyperinflated (TLC > 120% pred. or RV/TLC > 0.4 and RV > 140% pred.). Of participants with active asthma, 49 (46%) had RV pred. > 120%, 11 (10% had TLC pred. > 120%, 59 (56%) had RV/TLC ratio > 0.4 and 31 (29%) had DLCO < 80% predicted. We found no statistical association between hyperinflation and GINA symptom control, GINA treatment level, sex or being never smoker (Table 2).

**Table 2:** Comparison of factors associated with hyperinflation (TLC > 120% predicted, RV > 140% or RV/TLC ratio > 0.4) for persons with active asthma

	Odds ratio (95% CI)		*P*-value
**Hyperinflation (*N* = 25/103)**			
Asthma severity (Gina step 1 reference)	Gina 2–3	Gina 4–5	0.1052
	2.60 (0.79—8.53)	3.25 (1.06—10.30)	
Gina control (well-controlled reference)	Partially controlled	Uncontrolled	0.9773
	0.97 (0.36—2.62)	0.86 (0.21—3.52)	
Female sex	0.88 (0.35—2.18)		0.7766
Ever smoking	1.07 (0.43—2.64)		0.8828

Numbers from 106 patients with active asthma at follow-up. TLC, RV and sR_aw_ 3 missing

*OR* odds ratio

### Diffusion capacity

No significant differences were seen in the total diffusing capacity for carbon monoxide between the patients with active asthma (DLCO 87.7% predicted, 95% CI 84.3 – 91.1) and those in remission (DLCO 84.7% predicted, 95% CI 78.9 – 90.4) (*P* = 0.357). However, when adjusted for alveolar volume, the comparison showed higher diffusion capacities among the patients having active asthma (KCO 100% predicted, 95% CI 97. 2 – 103.6) compared with participants with asthma in remission (KCO, 93.9% predicted, 95% CI 88.8 – 99.0) (*p* = 0.102). When adjusted for age and smoking (number of pack-years), we found DLCO with an increase of 0.53% (95% CI 0.19 – 0.87) per year with asthma (*p* = 0.003). When adjusted for age, smoking and volume, we found that longer asthma duration was associated with higher KCO with an increase of 0.47% (95% CI 0.15 – 0.80) per year with asthma (*p* = 0.005).

We found that patients with partially controlled (ΔDLCO –8.17, 95% CI –15.7 to 0.62) and uncontrolled (ΔDLCO –14.3, 95% CI –14.3 to –5.20) asthma had lower DLCO than patients with well-controlled disease (Table 3), but this difference was not found for KCO.

**Table 3:** Comparison of associations between static lung volumes and diffusion capacity, and patients with controlled, partially controlled and uncontrolled asthma

Variables	Well-controlled	Partially controlled	*P*-value	Uncontrolled	*P*-value
TLC% pred	1.00	Δ-1.16 (-6.59—4.27)	0.6726	Δ-4.13 (-10.7—2.48)	0.2182
DLCO%, pred	1.00	Δ-8.17 (-15.7 to -0.62)	0.0343	Δ-14.3 (-23.4 to -5.20)	0.0024
KCO%,pred	1.00	Δ-4.85 (-12.2—2.48)	0.1925	Δ-3.83 (-12.7—5.03)	0.3931
RV % pred., estimate	1.00	Δ-2.39 (-14.4—9.68)	0.6955	Δ6.17 (-8.53—20.9)	0.4072
RV/TLC ratio, OR > 0.4	1.00	0.47 (0.19—1.15)	0.0390	Δ1.14 (0.38—3.54)	0.2934
sR_aw_ % pred	1.00	Δ10.1 (-32.1—52.3)	0.6353	Δ71.7 (19.5—123.8)	0.0076

Numbers from 106 patients with active asthma at follow-up. TLC, RV and sR_aw_ 3 missing. DLCO 5 missing

*OR* odds ratio

### Airway resistance

Total airway resistance sR_aw_, expressed as a percentage of predicted value, was higher among patients with active asthma (sR_aw_/proc 145.7% predicted, 95% CI 126.8 –164.7) than those in remission (117.0% predicted, 95% CI 79.7 – 154.3).

Patients classified as having GINA step 4 to 5 asthma (ΔRV 14.8%, pred., 95% CI 2.61 – 27) had higher residual volumes and higher airway resistance compared with patients with mild asthma (ΔRV 53% pred., 95% CI 9 – 97) (Table 4). The analyses also revealed that patients with uncontrolled disease (ΔsR_aw_ 71.7% pred., 95% CI 19.5 – 123.8) had higher airway resistance than partly controlled participants (ΔsR_aw_ 10.1% pred., 95% CI -32.1 – 52.3). Patients with only partially controlled disease were more hyperinflated than patients with well-controlled disease.

**Table 4:** Comparison of associations between static lung volumes and diffusion capacity, and patients with mild, moderate, and severe asthma

Variables	Mild asthma	Moderate asthma (GINA step 2–3)	*P*-value	Severe asthma (GINA step 4–5)	*P*-value
TLC% pred	1.00	Δ3.10 (-2.68—8.88)	0.2899	Δ2.23 (-3.37—7.84)	0.4314
DLCO%, pred	1.00	Δ5.22 (-3.12—13.6)	0.2168	Δ1.15 (-7.02—9.32)	0.7806
KCO%, pred	1.00	Δ1.71 (-6.12—9.54)	0.6659	Δ2.18 (-5.49—9.85)	0.5744
RV % pred	1.00	Δ6.40 (-6.17—19.0)	0.3147	Δ14.8 (2.61—27.0)	0.0178
RV/TLC ratio, OR > 0.4	1.00	1.28 (0.49—3.30)	0.9108	1.47 (0.59—3.66)	0.5389
sR_aw_ % pred	1.00	Δ13.6 (-32.0—59.3)	0.5546	Δ53.3 (9.06—97.6)	0.0187

Numbers from 106 patients with active asthma at follow-up. TLC, RV and sR_aw_ 3 missing. DLCO 5 missing

*OR* Odds ratio

## Discussion

This crosssectional study of 125 adults with asthma showed that patients with active asthma were more hyperinflated than patients with asthma in complete remission. Similarly, patients with more severe disease also had higher residual volume and patients with poorly controlled asthma were also more hyperinflated. We found that longer asthma duration was associated with higher diffusion capacity when adjusted for age and smoking. Finally, the levels of airway resistance were higher among patients with active, severe, or uncontrolled asthma compared with those in remission, with mild disease or well-controlled asthma.

It has been reported previously that hyperinflation is often observed in patients having active asthma [32]. Kharevic et al. [33] found RV levels and RV/TLC ratios significantly higher in their cohort of 31 patients with severe asthma compared with 23 patients with mild to moderate asthma, and by that more pronounced hyperinflation. These results align with our findings of a higher degree of RV in patients with severe asthma. However, we lacked the power to show a statistically significant association with hyperinflation among patients with severe asthma [11–15]. Our finding that airway resistance in patients with severe or uncontrolled asthma was higher than predicted correlates with previous studies [34]. Scichilone et al. [35] likewise found significantly higher ratios of residual volume/total lung capacity (RV/TLC). Therefore, they suggested that structural and functional changes of peripheral airways contribute to the pathogenesis of severe asthma. In a study by Sorkness et al. [36] of 287 patients with severe asthma and 382 patients with non-severe asthma, they found that RV/TLC was significantly higher in the former at any given level of airway obstruction as expressed by FEV_1_/FVC ratio. The importance of understanding hyperinflation in asthma has been recently illustrated by van der Meer et al. [4] who showed that hyperinflation negatively impacts overall health and activity. This cohort of individuals with well-defined asthma showed a large proportion had hyperinflation; as hyperinflation potentially can be alleviated [37], there is a need for awareness of this aspect of the disease.

We found higher diffusion capacities among patients with active asthma. There was no difference in diffusion capacity when comparing different degrees of asthma severity. Collard et al. [38] also found that diffusing capacities are either normal or high among never smokers with asthma; they reported that elevated diffusion capacities might be attributed to better perfusion of the apices of the lungs due to increased pulmonary arterial pressure or more negative pleural pressure because of bronchial narrowing. The increased diffusing capacities found among patients with severe active asthma could also be explained by hyperinflation and increased intrathoracic pressure. Finally, increased pulmonary capillary blood volume or extravasation of red blood cells in the alveolus also may contribute [39]. Furthermore, patients with asthma have been noted to have increased airway vascularization and, therefore, a larger capillary surface area of the lungs [40].

Graham et al. [41] suggested that high levels of DLCO in asthma partly is due to overestimation of DLCO due to airflow limitation. Weitzman and Wilson et al. [42] found elevated diffusing capacities in most of their cohort of asthma patients, which they attributed to greater perfusion of the apices of the lungs in patients with asthma. Likely the mechanism is a combination of all these mechanisms. Our finding that a longer asthma duration is associated with higher DLCO, which fits with a longer period of airway re-modelling resulting in the above-mentioned changes.

### Strengths and limitations

One of the strengths of this study is that it is a well-characterized cohort of patients diagnosed with asthma based both on history and objective findings and evaluated by a respiratory physician. However, it is a limitation that relatively many of the patients examined at baseline for different reasons did not participate in the follow-up examination. Another limitation, although primarily caused by lack of access to equipment, is that the patients at baseline did not have their static lung volumes and diffusion capacity measured. We do not know the duration of the participant’s remission and can therefore not examine the impact on differences in diffusion capacity and hyperinflation.

In conclusion, this cross-sectional study shows that asthma is associated with a high DLCO and that longer asthma duration is associated with increased diffusing capacities after adjusting for age and smoking. Furthermore, our findings reveal a possible close interaction between airflow limitation, air trapping, and increased diffusing capacity among patients with long-standing active asthma. As a substantial proportion of patients with asthma are hyperinflated, there should be a greater awareness of this aspect in daily clinical practice.

## Data Availability

The data are available upon reasonable request, but analysis may require approval from the regional data safety committee for the capital region of Denmark (Videnscenter for dataanmeldelser).
